# Covert Information Mapped Generalized Spatial and Direction Modulation toward Secure Wireless Transmission

**DOI:** 10.3390/s24196333

**Published:** 2024-09-30

**Authors:** Yuan Zhong, Zhengyu Ji, Xianglu Li, Peng Fei, Dong Hou, Zhigang Wang, Jie Tian

**Affiliations:** 1 Institute of Electronic Engineering, China Academy of Engineering Physics, Mianyang 621999, China; 2 Key Laboratory of Science and Technology on Communications, University of Electronic Science and Technology of China, Chengdu 611731, China; 3 The First School, Rocket Force University of Engineering, Xi’an 710025, China; 4 Guangdong Communications and Networks Institute, Guangzhou 510289, China

**Keywords:** spatial and directional modulation (SDM), generalized spatial modulation (GSM), covert information mapping (CIM), receiver subset selection (RRS), eavesdropping

## Abstract

In this paper, for the sake of enhancing the security of wireless transmission, we proposed a novel system based on spatial and direction modulation (SDM) combined with generalized spatial modulation (GSM) which is aided by covert information mapping (CIM), termed as the CIM-GSDM system. In such a system, the legitimated user is equipped with distributed receivers so as to demodulate the conveyed signal by exploiting its indices while disturbing eavesdroppers for information security. More specifically, part of the information is modulated into the indices of the legitimated distributed receiver subsets with the aid of the mapped covert information and the interference matrix, while another part of the message is arranged by conventional amplitude-phase modulation. The proposed system can reap the benefits from both GSM and CIM to make eavesdropper suffer great mixture. Furthermore, the detection scheme and theoretical analysis of error performance are discussed as well. The simulation results exhibit that the bit error rate (BER) performance of legitimate user is much better than that of the eavesdropper while the proposed scheme improves the security compared to the original CIM-SDM system at the same spectral efficiency.

## 1. Introduction

Due to the rapid development of communications, mobility adaptation has shown its importance in modern communication systems. In this case, the transmit signal is exposed in the wireless environment, which leads to severe security problems due to the lack of physical layer security (PLS) [1]. In order to gain effective security protection through wireless transmission, directional modulation (DM) has been proposed [2,3,4] which exploits spatial characteristics of the transmitter (Alice), legitimate receiver (Bob), and eavesdropper (Eve), so as to transmit information to Bob while interfering Eve. Unfortunately, although DM has been widely researched, it is unwise to adopt DM directly if Eve happens to locate in the same receive direction as Bob [5,6]. Because information can be extracted by Eve. Bob has no more obvious advantages in such a configuration, leading to the loss of wireless security [7,8].

For the sake of further improving the achievable security, spatial and directional modulation (SDM) was designed with the aid of DM and spatial modulation (SM) [9], attempting to enhance the security weakened by the aforementioned traditional schemes. By utilizing multiple distributed receivers, it can not only convey part of information using the index of the activated receiver at Bob, but also keep considerable transmission security offered by DM [10,11]. Therefore, SDM is capable to provide improved transmission security where some information is concealed in the receiver indices. However, Eve will make every effort to achieve eavesdropping, then the security enhancement will suffer potential challenge whenever Eve performs numerous distributed receivers and some of them are close to Bob’s [12].

Recently, Refs. [13,14] constituted an attractive security-enhanced scheme that combines SDM with covert information mapping (CIM) in the context of distributed receivers, called CIM-SDM, as integrating the idea of secret modulation to SDM. On the one hand, SDM can hide the information in the receiver indices. On the other hand, CIM is able to insert the covert information into the space-time antenna index mapping with a careful design and make Eve suffer from severe interference [15]. Hence, significant improvement of transmission security can be achieved by this approach, even if Eve has exactly the same multiple receiver distribution as Bob. Nevertheless, though SDM can prevent eavesdropping validly, it has an unavoidable limitation in low spectral efficiency due to activating only one receiver at a time.

Moreover, the work of [16] has conceived a novel generalized spatial modulation (GSM) scheme for SM systems. Instead activating one receiver at a time, GSM can deliver multiple symbols by activating multiple activated receivers simultaneously [16,17] while the conveyed information is hidden in indices of receiver subsets leading to a hybrid of SM and spatial multiplexing [18,19]. The application of GSM will increase the spectral efficiency and rate effectively and it can improve the transmission obviously with the rising of the scale of distributed receivers [20,21]. From a cross-layer point of view, it is appealing to combine GSM and SDM for making full use of both advantages.

Against this backdrop, in this paper, we aim to enhance the security of transmission while improving transmission rate efficiently by jointly considering current CIM-SDM and GSM (CIM-GSDM), which is realized by the interference factor with the assistance of embedding the covert information, as well as concealing indices of receivers subsets, where multiple eavesdroppers exist. In particular, SDM and GSDM are both based on direction modulation, but SDM activates only one receiver in each transmission while GSDM can activate multiple receivers simultaneously which improves the transmission rate. In this scenario, CIM-GSDM can enhance transmission security effectively at Bob while decreasing unexpected eavesdropping at Eve simultaneously. Specifically, the basic model of GSDM will be described at first, then integrated by CIM to finish the whole structure building. What is more, the theoretical analysis of the average bit error rate (ABER) at both Bob and Eve are exhibited for clearer performance comparison. Finally, the simulation results are presented to investigate the benefits of the proposed scheme, even if Eve is equipped with the identical distributed receivers as Bob’s, which supports the theoretical analysis.

The rest of this paper is organized as follows. Section 2 describes the system model used in this paper thoroughly, which is constructed by GSDM in conjunction with CIM. In Section 3, the theoretical analysis of ABER at both Bob and Eve are derived for further comparison. Our simulation results and performance analysis are presented in Section 4 and we conclude our discourse in Section 5 finally.

*Notation:* In this paper, ${\left(\cdot \right)}^{H}$ and $∥\cdot ∥$ represent the conjugate transpose of a matrix and the Frobenius norm, respectively. What is more, $R\left(\cdot \right)$ indicates the real operators and CN denotes circularly symmetric complex Gaussian distribution.

## 2. Convert Information Mapping Aided GSDM

**Figure 1:**
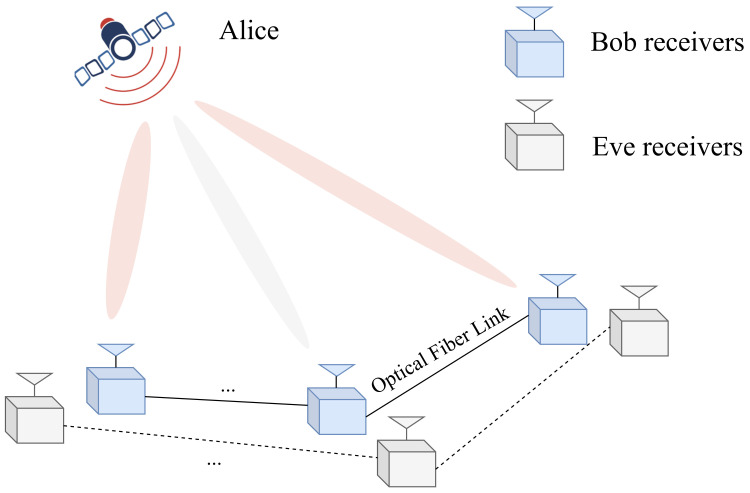
The system of generalized spatial and direction modulation.

As shown in Figure 1, a directional modulation system which transmits in free space is considered in this paper. The transmitter Alice is equipped with $N_{t}$ antennas and the legitimate Bob is equipped with $N_{r}$ cooperative single-antenna receivers, which are located in different spatial directions and are linked through optical fibers. In addition, there are $N_{u}$ receivers at Bob activated in communication where $N_{u}<N_{r}$. That is to say, only part of receivers at Bob are participated in transmission. Meanwhile, it is assumed that Eve is equipped with $N_{e}$ cooperative single-antenna receivers linked through optical fibers as well, which sends nothing to hide itself and try its best to eavesdrop. In this section, the GSDM system will be introduced at first and followed by the proposed CIM-GSDM system in details.

### 2.1. The GSDM System Model

In order to take both transmission rate and security performance into consideration, one of functions of GSM, receiver subset selection (RRS), is introduced to SDM where multiple eavesdroppers exist. Based on RSS, in such a case that Bob is equipped with $N_{r}$ receivers and only $N_{u}$ of them are activated, the number of total single-antenna receivers combinations is $C_{Nr}^{Nu}$. For the sake of convenience for information mapping, the number of receivers combinations chosen to use should be the power of two. Hence, there are $f_{1}=2^{\left\lfloor \log_{2}C_{N_{r}}^{N_{u}}\right\rfloor }$ combinations allowed for modulation and $k_{1}=\log_{2}f_{1}$ bits are delivered by RRS. Apart from information carried by indices of receiver subsets, $N_{u}$ receivers are activated and each of them is able to deliver single *M*-ary amplitude-phase modulation (APM) symbol. Therefore, other $k_{2}=N_{u}\times \log_{2}M$ bits are transmitted by conventional APM symbols though $N_{u}$ activated receivers. As a result, the GSDM system is capable to convey $k=k_{1}+k_{2}$ bits a time so the transmission rate is improved efficiently.

For simplicity, assume that Alice is equipped with uniform linear array (ULA), where the phase center is the geometric center of array. Then the channel vector between Alice and the receiver located in direction of angle θ is expressed as

(1) $$h^{H}\left(\theta \right)=\left[e^{-j\left(\frac{N_{t}-1}{2}\right)\frac{2\pi }{\lambda }d\cos\theta },e^{-j\left(\frac{N_{t}-1}{2}-1\right)\frac{2\pi }{\lambda }d\cos\theta },\ldots ,e^{j\left(\frac{N_{t}-1}{2}\right)\frac{2\pi }{\lambda }d\cos\theta }\right],$$

where λ denotes the signal wavelength and d≤λ/2 is the antennas spacing at Alice. Thus, the channel matrix between Alice and Bob can be expressed as

(2) $$H\left(\Theta_{B}\right)={\left[h\left(\theta_{1}\right),h\left(\theta_{2}\right),\ldots ,h\left(\theta_{N_{r}}\right)\right]}^{H},$$

where $H\left(\Theta_{B}\right)\in C^{N_{r}\times N_{t}}$, $\Theta_{B}=\left\{\theta_{1},\theta_{2},\ldots ,\theta_{N_{r}}\right\}$ and $h\left(\theta_{i}\right),i=1,2,\ldots ,N_{r}$ represent the set of directional angles at Bob and the channel vector between Alice and *i*-th receiver at Bob, respectively.

For the purpose of transmission security, the beamforming vector should provide the maximum power to Bob. Then the corresponding beamforming vector $w_{i}$ to *i*-th receiver at Bob is given by

(3) $$w_{i}=h\left(\theta_{i}\right)/N_{t}.$$

When taking all receivers at Bob into consideration, the beamforming matrix $W\in C^{N_{t}\times N_{r}}$ of the channel can be represented by

(4) $$W=\left[w_{1},w_{2},\ldots ,w_{N_{r}}\right].$$

As aforementioned, $k=k_{1}+k_{2}$ bits can be modulated at a time. More specifically, symbol vector $x_{l,s}$ produced by Alice can be formulated as

(5) $$x_{l,s}=I_{l}b_{s},$$

where $I_{l}\left(l=1,2,\ldots ,2^{k_{1}}\right)$ denotes matrix which consists of $N_{u}$ column vector chosen from identity matrix $I_{N_{r}\times N_{r}}$, as $N_{u}$ activated receivers. In addition, $b_{s}\left(s=1,2,\ldots ,2^{k_{2}}\right)$ is a column vector built up by $N_{u}$ symbols selected from conventional APM symbols. It is noteworthy that the symbol vector $x_{l,s}$ involves information of activated receiver subset in conjunction with APM symbols which both are able to influence the performance.

For the sake of security enhancement, an interference matrix is proposed here. The interference matrix is known to Bob but unknown to Eve. Therefore, the transmit signal at Alice can be detailed as

(6) $$s_{l,s}=W\Lambda x_{l,s},$$

where $\Lambda =\mathrm{diag}\left(\lambda_{1},\lambda_{2},\ldots ,\lambda_{N_{r}}\right)\in C^{N_{r}\times N_{r}}$ is interference matrix and $\lambda_{i}=e^{j\varphi_{i}},i=1,2,\ldots N_{r}$ denotes interference factors. Every receiver at Bob has a corresponding interference factor, which is relatively big for efficient phase disturbance. Moreover, Bob can set different interference factors to prevent eavesdropping of single legitimate receiver at Bob for further security.

Transmitting through the channel in free space, the receive signal at Bob can be represented as

(7) $$\begin{array}{cc} r_{B} & =H\left(\Theta_{B}\right)s_{l,s}+n_{B}\\ \; & =H\left(\Theta_{B}\right)W\Lambda x_{l,s}+n_{B}. \end{array}$$

With regards to Eve, the receive signal can be expressed as

(8) $$\begin{array}{cc} r_{E} & =H\left(\Theta_{E}\right)s_{l,s}+n_{E}\\ \; & =H\left(\Theta_{E}\right)W\Lambda x_{l,s}+n_{E}, \end{array}$$

where $H\left(\Theta_{E}\right)\in C^{N_{e}\times N_{t}}$ denotes the channel matrix between Alice and Eve, $\Theta_{E}=\left\{\theta_{1},\theta_{2},\ldots ,\theta_{N_{e}}\right\}$ is the set of directional angles at Eve. Additionally, $n_{B}$ and $n_{E}$ are noise vectors to Bob and Eve, which obey $n_{B}∼CN\left(0,\sigma_{B}^{2}I_{N_{r}\times N_{r}}\right)$ and $n_{E}∼CN\left(0,\sigma_{E}^{2}I_{N_{o}\times N_{s}}\right)$, respectively. According to [13], the diagonal elements of matrix $H\left(\Theta_{B}\right)W$ are all equal to 1 and diagonal elements of matrix $H\left(\Theta_{E}\right)W$ are all less than or equal to 1, which indicates the amplitude or power of the receive signal at Eve decreases. Hence, it is undeniable that the receive signal at Eve are extremely disturbed while Bob can receive successfully as usual, so the security of transmission gains great enhancement.

### 2.2. Proposed CIM-GSDM Scheme

For the sake of further improvement, the GSDM system is able to combine with CIM in which covert information is mapped with a deliberate design in the GSDM system. In the proposed CIM-GSDM scheme, the non-covert information and the covert information are considered together at Alice. The proposed scheme are still based on the GSDM system, and the difference and novelty lie in that the information delivered by receiver subsets are not only designed by indices, but also the covert information added in. Since the covert information is related to the past message, Bob can demodulate correctly owing to knowing the rule while Eve falls into confusion from the beginning. Therefore, it is distinct to the GSDM system and improve the security of transmission to a next level in the presence of CIM.

Under this scenario, the data bits to send at a time are separated into three parts, as $k=k_{1}+k_{2}+k_{3}$, which are exploited to design as the covert message and APM symbols. Specifically, $k_{1}+k_{2}$ bits are employed as selection of receiver subset indices with the aid of CIM design and remaining $k_{3}$ bits are applied for APM symbols. Based on CIM, all receivers are divided into two groups $I_{1}$ and $I_{2}$, in which $I_{1}=\left\{1,2,\cdots ,\frac{N_{r}}{2}\right\}$ and $I_{2}=\left\{\frac{N_{r}}{2}+1,\frac{N_{r}}{2}+2,\cdots ,N_{r}\right\}$ include the former half receivers and the latter half receivers, respectively. In this case, there are only $f_{2}=2^{\left\lfloor \log_{2}C_{N_{r}/2}^{N_{u}}\right\rfloor }$ receiver combinations in each group at Bob with $k_{2}=\log_{2}f_{2}$. Thus, $2f_{2}$ groups can be modulated in total. The main idea of CIM-GSDM is to use $k_{1}$ bits to choose the receiver group in conjunction with message at last slot and use $k_{2}$ bits to select the receivers combination index in the group. In detail, if $K_{1}=\left[1\right]$, the current receiver group is the same as the previous one. Otherwise, the current receiver group is different from the previous one if $K_{1}=\left[0\right]$, that is to say, choosing another receiver group. Supposing that the receiver group index selected to modulate at the (k−1)-th slot is $I_{1}$, the receiver group index $I_{gk}$ chosen at the *k*-th slot can be represented as

(9) $$I_{g_{k}}=\left\{\begin{array}{cc} I_{2}, & \;\mathrm{if}\;K_{1}=\left[0\right]\;\mathrm{and}\;I_{g_{k-1}}=I_{1}\\ I_{1}, & \;\mathrm{if}\;K_{1}=\left[1\right]\;\mathrm{and}\;I_{g_{k-1}}=I_{1}. \end{array}\right.$$

Analogously, if the receiver group index selected to modulate at (k−1)-th slot is $I_{2}$, then $I_{gk}$ can be represented as

(10) $$I_{g_{k}}=\left\{\begin{array}{cc} I_{1}, & \;\mathrm{if}\;K_{1}=\left[0\right]\;\mathrm{and}\;I_{g_{k-1}}=I_{2}\\ I_{2}, & \;\mathrm{if}\;K_{1}=\left[1\right]\;\mathrm{and}\;I_{g_{k-1}}=I_{2}. \end{array}\right.$$

**Figure 2:**
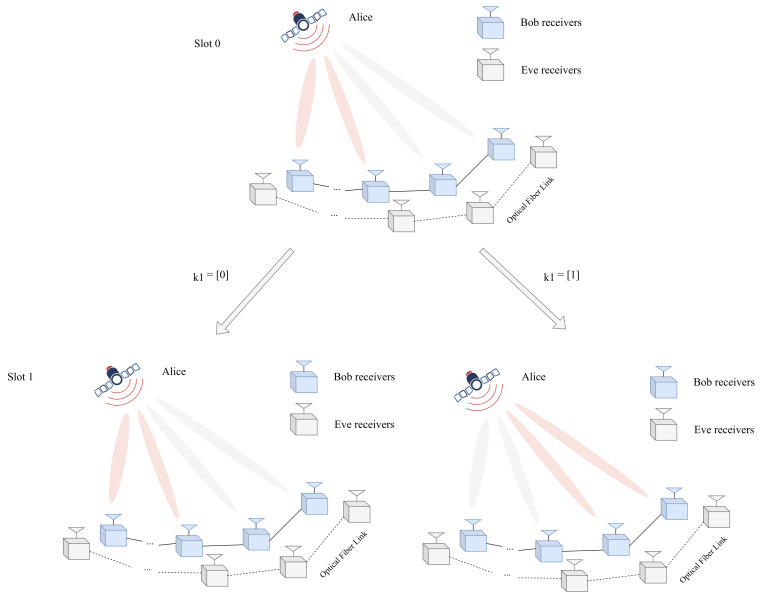
An example with $N_{r}=4$ for the proposed CIM-GSDM scheme.

For instance, an example with $N_{r}=4$ for the proposed CIM-GSDM scheme is shown in Figure 2. In a nutshell, the design of selection for receiver groups at Bob takes the message of the former and the latter into consideration together and provide further security. After the receiver group is fixed, $k_{2}$ bits are employed to determine the receive subset index in the certain group $I_{g_{k}}$ as $I_{g_{k}}\left(p\right)$,$\left(p=1,2,\ldots ,f_{2}\right)$. Then the symbol vector produced by Alice can be expressed as

(11) $$x_{I_{g_{k}}}^{s}=I_{I_{g_{k}}}b_{s},$$

where $I_{I_{g_{k}}}$ consists of $N_{u}$ column vector chosen from identity matrix $I_{N_{r}\times N_{r}}$ corresponding to $I_{g_{k}}$, $b_{s}\left(s=1,2,\ldots ,2^{k_{3}}\right)$ is a column vector built up by $N_{u}$ symbols selected from conventional *M*-ary APM symbols. Therefore, the transmit signal at Alice can be represented as

(12) $$s_{I_{g_{k}}}^{s}=W\Lambda x_{I_{g_{k}}}^{s}.$$

Furthermore, the transmission rate of the CIM-GSDM system can be formulated as

(13) $$R_{p}=\log_{2}2f_{2}+N_{u}\log_{2}\left(M\right)\;\left(\mathrm{bpcu}\right).$$

In general, due to the covert information mapped in transmission, Eve suffers extremely interference even if receivers at Eve are located in exactly the same direction as Bob’s. The reason why the proposed system is capable to hold such reliable transmission is that Eve has no idea about the covert information related to the last slot so the message it can catch is only the current one. In addition, keeping the interference matrix is beneficial to enhance the security of transmission as well. Thus, until now, employment of CIM-GSDM has at least two layers of protection for security of transmission as improving the transmission rate and spectral efficiency obviously, which gains considerable enhancement compared to the original SDM system as a novelty.

### 2.3. Detection Algorithm for the Proposed Scheme

Based on (12), the receive signal at Bob aided by CIM at the *k*-th slot can be expressed as

(14) $$\begin{array}{cc} r_{B}^{k} & =H\left(\Theta_{B}\right)s_{I_{g_{k}}}^{s}+n_{B}\\ \; & =H\left(\Theta_{B}\right)W\Lambda x_{I_{g_{k}}}^{s}+n_{B}. \end{array}$$

Assume that the optimal detector of maximum likelihood (ML) is adopted to detect the composition of unit column vectors corresponding to the receiver subset *l* and the combination of APM symbols *s*, which can be formulated as

(15) $$\langle \hat{l},\hat{s}\rangle =\arg\min_{\hat{l}\in L,s\in S}\left\{{∥r_{B}^{k}-H\left(\Theta_{B}\right)W\Lambda x_{I_{g_{k}}}^{s}∥}^{2}\right\},$$

where $L=\left\{1,2,\ldots ,2f_{2}\right\}$ and $S=\left\{1,2,\ldots ,2^{k_{3}}\right\}$ denotes the set of the indices of receivers subsets at Bob and the set of constellations combinations of *M*-ary APM symbols, respectively.

Furthermore, the index of receiver group can be set as

(16) $${\hat{g}}_{k}=\left\{\begin{array}{cc} 0, & \;\mathrm{if}\;\hat{l}\leq f_{2}\\ 1, & \;\mathrm{if}\;\hat{l}>f_{2}. \end{array}\right.$$

In particular, the bit $K_{1}$ for determining the receive group index is decided by ${\hat{g}}_{k}$ and ${\hat{g}}_{k-1}$, which can be given by

(17) $$K_{1}=\left\{\begin{array}{cc} 1, & {\hat{g}}_{k}={\hat{g}}_{k-1}\\ 0, & {\hat{g}}_{k}\neq {\hat{g}}_{k-1}. \end{array}\right.$$

In addition, the index of receiver subset $\hat{p}$ in the receive group $I_{g_{k}}$ can be detailed as follows:

(18) $$\hat{p}={\mathrm{MOD}}_{f_{2}}\left(\hat{l}-1\right)+1,$$

where ${\mathrm{MOD}}_{a}\left(b\right)$ returns the remainder after division of *b* by *a*. As a result, $\hat{p}$ can be exploited to demodulating the index bits $k_{2}$ of the receive subset.

With respect to Eve, the receive signal at Eve at the *k*-th slot can be expressed as

(19) $$\begin{array}{cc} r_{E}^{k} & =H\left(\Theta_{E}\right)s_{I_{g_{k}}}^{s}+n_{E}\\ \; & =H\left(\Theta_{E}\right)W\Lambda x_{I_{g_{k}}}^{s}+n_{E}. \end{array}$$

Eve is possible to obtain channel state information (CSI) of the system and achieves to calculate the beamforming matrix *W*. However, the interference matrix is still hard to confirm. As a result, the ML detector employed at Eve can be represented as

(20) $$\langle \hat{l},\hat{s}\rangle =\arg\min_{\hat{l}\in L,s\in S}\left\{{∥r_{E}^{k}-H\left(\Theta_{E}\right)W\Lambda x_{I_{g_{k}}}^{s}∥}^{2}\right\}.$$

In general, the CIM-GSDM system are much more safe than the original system. On the one hand, only part of the disturbed receivers at Bob are activated and adjusted by the covert information, which is hard to be ensured at Eve. On the other hand, due to the interference matrix added based on (19) and (20), the accuracy of detection performance of APM symbols suffers reduction significantly. That is to say, except to $k_{1}$ and $k_{2}$ bits, Eve can hardly obtain $k_{3}$ bits neither. By contrast, Bob is able to demodulate all the bits successfully since Bob knows the rules. As a benefit, the proposed strategy of CIM-GSDM can let Alice communicate with Bob smoothly while disturbing Eve, which enhances the security of transmission effectively.

## 3. Performance Analysis

In this section, the theoretical upper bound of ABER at both Bob and Eve in the proposed CIM-GSDM system over free space channels will be derived and analyzed, which jointly considers the influence of mapped covert information and disturbed receivers subset selection.

### 3.1. Bob’S Average Bit Error Probability

Based on assumption of (15), the upper bound of ABER at Bob can be formulated as

(21) $$P_{B_{k}}⩽\frac{1}{R_{p}2^{R_{p}}}\sum_{x_{I_{g_{k}}}^{s}\in X}\sum_{x_{I_{m_{k}}}^{n}\in X\neq x_{I_{g_{k}}}^{s}}d\left(x_{I_{g_{k}}}^{s}\to x_{I_{m_{k}}}^{n}\right)P\left(x_{I_{g_{k}}}^{s}\to x_{I_{m_{k}}}^{n}\right),$$

where $R_{p}$ denotes the transmission rate based on (13), $d\left(x_{I_{g_{k}}}^{s}\to x_{I_{m_{k}}}^{n}\right)$ represents the Hamming distance between vectors $x_{I_{g_{k}}}^{s}$ and $x_{I_{m_{k}}}^{n}$, $P\left(x_{I_{g_{k}}}^{s}\to x_{I_{m_{k}}}^{n}\right)$ is the pairwise Error probability (PEP).

In particular, the PEP in (21) can be given by

(22) $$\begin{array}{c} P\left(x_{I_{g_{k}}}^{s}\to x_{I_{m_{k}}}^{n}\right)\\ =P\left({∥r_{B}^{k}-H_{\Lambda }x_{I_{g_{k}}}^{s}∥}^{2}>{∥r_{B}^{k}-H_{\Lambda }x_{I_{m_{k}}}^{n}∥}^{2}\right)\\ =P\left\{R\left[{n_{B}}^{H}\left(H_{\Lambda }x_{I_{m_{k}}}^{n}-H_{\Lambda }x_{I_{g_{k}}}^{s}\right)\right]>\frac{1}{2}{∥H_{\Delta }x_{I_{m_{k}}}^{n}-H_{\Lambda }x_{I_{g_{k}}}^{s}∥}^{2}\right\}, \end{array}$$

where $H_{\Lambda }=H\left(\Theta_{B}\right)W\Lambda$ is defined and $n_{B}∼CN\left(0,\sigma_{B}^{2}I_{N_{r}\times N_{r}}\right)$.

Noting that $R\left[{n_{B}}^{H}\left(H_{\Lambda }x_{I_{m_{k}}}^{n}-H_{\Lambda }x_{I_{g_{k}}}^{s}\right)\right]$ is a Gaussian random variable obeying $\mathrm{CN}∼\left(0,\frac{1}{2}\sigma_{n}^{2}{∥H_{\Lambda }\left(x_{I_{g_{k}}}^{s}-x_{I_{m_{k}}}^{n}\right)∥}^{2}\right)$, PEP can be obtained as

(23) $$P\left(x_{I_{g_{k}}}^{s}\to x_{I_{m_{k}}}^{n}\right)=Q\left(\sqrt{\frac{{∥H_{\Lambda }\left(x_{I_{g_{k}}}^{s}-x_{I_{m_{k}}}^{n}\right)∥}^{2}}{2\sigma_{n}^{2}}}\right).$$

After that, the upper bound of ABEP of Bob at the *k*-th slot can be obtained by substituting (23) to (21). However, the signal conveyed at Bob is associated with the mapped covert information and it will affect the practical ABEP. As a result, it should take influence of CIM into consideration jointly while calculating the upper bound of ABER.

Against this scenario, the influence of CIM should be considered in the analysis of ABEP. Noticing that the mapped covert information will not affect the Hamming distance and PEP between $x_{I_{g_{k}}}^{s}$ and $x_{I_{m_{k}}}^{n}$, but may influence mapping of the $k_{1}$ bits in which covert information embeds. According to (17), if the former $k_{1}$ bit is determined wrongly, the latter one is likely to be misjudged. In other words, the accuracy of the latter $k_{1}$ bit is depended on the former one in some extent. Therefore, the average error probability of the bit for receiver group selection can be approximated by:

(24) $$P_{r}=P_{B_{k-1}}\times \left(1-P_{B_{k}}\right)+\left(1-P_{B_{k-1}}\right)\times P_{B_{k^{\prime }}},$$

which illustrates that the misjudgment of the original $k_{1}$ bit is caused by the misjudgment of the receiver group at the (k−1)-th or *k*-th slot, even though the remaining data bits is correctly obtained. It can be used that $P_{B_{k-1}}=P_{B_{k}}$, (24) can be arranged as

(25) $$P_{r}=2P_{B_{k}}\left(1-P_{B_{k}}\right).$$

Furthermore, the ABEP should be adjusted as the influence of the mapped covert information discussed aforementioned, which is considered the (k−1)-th slot and the *k*-slot jointly and can be detailed as

(26) $$\begin{array}{cc} {\bar{P}}_{B} & =\frac{1}{R_{p}}\times P_{r}+\frac{\left(R_{p}-1\right)}{R_{p}}\times P_{B_{k}}\\ \; & =\frac{\left(R_{p}+1-2P_{B_{k}}\right)}{R_{p}}. \end{array}$$

In a nutshell, the upper bound of ABEP at Bob is obtained at last.

### 3.2. Eve’s Average Bit Error Probability

Analogously, the upper bound of ABEP at Eve in the CIM-GSDM system at *k*-slot based on (20) can be formulated as

(27) $$P_{E_{k}}⩽\frac{1}{R_{p}2^{R_{p}}}\sum_{x_{I_{g_{k}}}^{s}\in X}\sum_{x_{I_{m_{k}}}^{n}\in X\neq x_{I_{g_{k}}}^{s}}d\left(x_{I_{g_{k}}}^{s}\to x_{I_{m_{k}}}^{n}\right)P_{H\left(\Theta_{E}\right)}\left(x_{I_{g_{k}}}^{s}\to x_{I_{m_{k}}}^{n}\right),$$

where $P_{H\left(\Theta_{E}\right)}\left(x_{I_{g_{k}}}^{s}\to x_{I_{m_{k}}}^{n}\right)$ denotes the PEP for Eve under the channel matrix $H\left(\Theta_{E}\right)$. In this scenario, the PEP in (27) is given by

(28) $$\begin{array}{c} P_{H\left(\Theta_{E}\right)}\left(x_{I_{g_{k}}}^{s}\to x_{I_{m_{k}}}^{n}\right)\\ =P\left({∥r_{E}^{k}-H_{E}x_{I_{g_{k}}}^{s}∥}^{2}>{∥r_{E}^{k}-H_{E}x_{I_{m_{k}}}^{n}∥}^{2}\right)\\ =P\left({∥H_{E}{\Lambda x}_{I_{g_{k}}}^{s}+n_{E}-H_{E}x_{I_{g_{k}}}^{s}∥}^{2}>{∥H_{E}{\Lambda x}_{I_{g_{k}}}^{s}+n_{E}-H_{E}x_{I_{m_{k}}}^{n}∥}^{2}\right)\\ =P\left(R\left[{n_{E}}^{H}\left(H_{E}x_{I_{m_{k}}}^{n}-H_{E}x_{I_{g_{k}}}^{s}\right)\right]>\frac{{∥H_{E}{\Lambda x}_{I_{g_{k}}}^{s}-H_{E}x_{I_{m_{k}}}^{n}∥}^{2}-{∥H_{E}{\Lambda x}_{I_{g_{k}}}^{s}-H_{E}x_{I_{g_{k}}}^{s}∥}^{2}}{2}\right), \end{array}$$

where $H_{E}=H\left(\Theta_{E}\right)W$ and $n_{E}∼CN\left(0,\sigma_{B}^{2}I_{N_{r}\times N_{r}}\right)$. Moreover, $R\left[{n_{E}}^{H}\left(H_{E}x_{I_{m_{k}}}^{n}-H_{E}x_{I_{g_{k}}}^{s}\right)\right]$ is a Gaussian random variable obeying $CN∼\left(0,\frac{1}{2}\sigma_{E}^{2}{∥H_{E}\left(x_{I_{g_{k}}}^{s}\to x_{I_{m_{k}}}^{n}\right)∥}^{2}\right)$. Therefore, the PEP for Eve can be formulated as

(29) $$P_{H\left(\Theta_{E}\right)}\left(x_{I_{g_{k}}}^{s}\to x_{I_{m_{k}}}^{n}\right)=Q\left(\frac{{∥H_{E}{\Lambda x}_{I_{g_{k}}}^{s}-H_{E}x_{I_{m_{k}}}^{n}∥}^{2}-{∥H_{E}{\Lambda x}_{I_{g_{k}}}^{s}-H_{E}x_{I_{g_{k}}}^{s}∥}^{2}}{\sqrt{2}\sigma_{E}∥H_{E}x_{I_{g_{k}}}^{s}-H_{E}x_{I_{m_{k}}}^{n}∥}\right).$$

By substituting (29) to (27), the theoretical upper bound of ABEP for Eve can be calculated. By contrast, Eve can not recognize the correct covert information and its corresponding bit since Eve has no idea about the rule of CIM. In this case, the average error probability of the bit for receiver group selection at Eve can be approximated by $P_{r}=0.5$. Specifically, the ABEP which is considered the (k−1)-th slot and the *k*-slot jointly can be detailed as

(30) $$\begin{array}{cc} {\bar{P}}_{r} & =\frac{1}{R_{p}}\times P_{r}+\frac{\left(R_{p}-1\right)}{R_{p}}\times P_{E_{k}}\\ \; & =\frac{0.5+\left(R_{p}-1\right)P_{E_{k}}}{R_{p}}. \end{array}$$

Finally, the upper bound of ABEP at Eve is obtained.

It should be noted that the theoretical bound of ABEP of Eve only can be attained in high SNR region due to its robust.

## 4. Simulation Results

In this section, we verify the simulation results and the theoretical performance of the proposed CIM-GSDM system in terms of Bob and Eve. Moreover, the comparison between the original CIM-SDM system and the proposed CIM-GSDM system is exhibited as well under the same spectral efficiency. More specifically, theoretical and simulated BER performance at both Bob and Eve is obtained and compared in details. In the process of simulations, assume that Alice is equipped with $N_{t}=8$ antennas and the distance between each antennas is d=λ/4. The signals are transmitted over free space channels. Considering Eve desires eavesdropping, it is assumed that the receivers at Eve are close to the Bob’s ones due to the characteristic of direction modulation. The interference matrix and the number of receivers at Bob and Eve are different in simulations. The assumptions are the same as theoretical analysis. More details about simulations are shown in Table 1 as follows.

**Figure 3:**
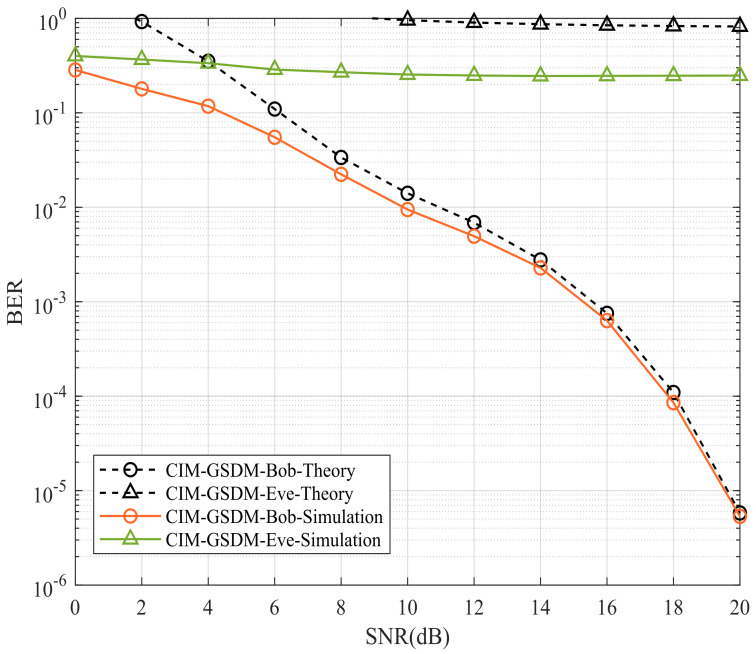
Bob’s and Eve’s BER performances of proposed CIM-GSDM scheme employing $N_{t}=8$, $N_{r}=6$, $N_{e}=6$ and QPSK.

As shown in Figure 3, Bob’s and Eve’s BER performances of proposed CIM-GSDM scheme employing $N_{t}=8$, $N_{r}=6$, $N_{e}=6$ and QPSK are compared. The interference matrix is $\Lambda =diag\left\{e^{j\Theta_{\Lambda }}\right\}\left(\Theta_{\Lambda }=\left\{28^{{}^{\circ}},32^{{}^{\circ}},45^{{}^{\circ}},55^{{}^{\circ}},60^{{}^{\circ}},65^{{}^{\circ}}\right\}\right)$. Observed in Figure 3, the theoretical curves of Bob both approximate well to the simulated curves at high signal noise radio (SNR). The BER performance of Bob are significantly better than that of Eve with the employment of the proposed CIM-GSDM system at both middle and high SNR even if the receivers at Eve are located near to Bob’s. The theoretical and simulation results perform better in high SNR region since the interference caused by noise relatively decreases with SNR increasing. More explicitly, it implies that the proposed CIM-GSDM system causes interference to Eve while achieves transmission to Bob, which develop the security of transmission as a benefit of employing the proposed CIM-GSDM scheme.

**Figure 4:**
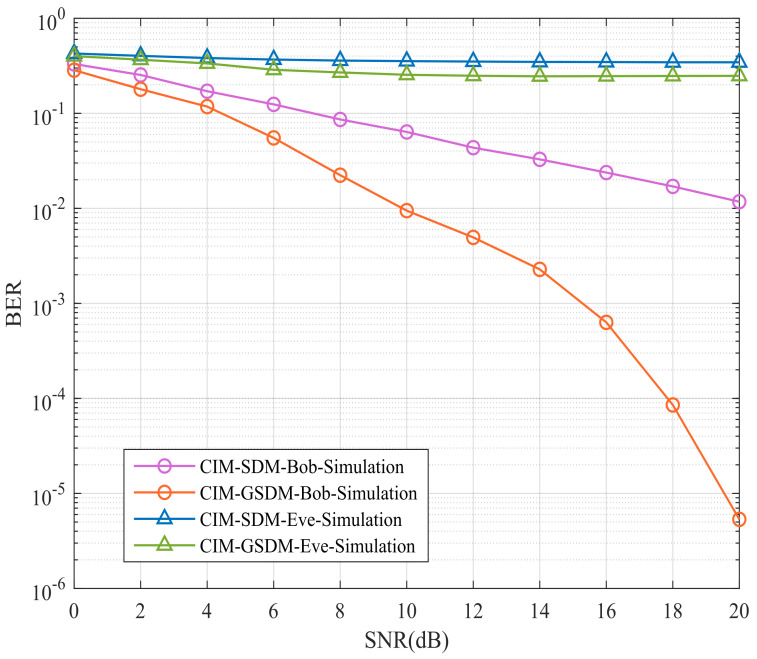
Bob’s and Eve’s BER performances of proposed CIM-GSDM scheme in comparison to the traditional CIM-SDM counterparts employing $N_{t}=8$, $N_{r}=6$, $N_{e}=6$ and QPSK at the same spectral efficiency.

Figure 4 suggests Bob’s and Eve’s BER performances of proposed CIM-GSDM scheme in comparison to the traditional CIM-SDM counterparts employing $N_{t}=8$, $N_{r}=6$, $N_{e}=6$ and QPSK at the same spectral efficiency. Specifically, the BER performance of Eve suffers great interference at both the CIM-SDM and CIM-GSDM scheme, which implies these two scheme can improve the security as it needs. Apart from this, another advantage ensured is the BER performance of Bob has much development in the proposed scheme than that of the original CIM-SDM scheme with nearly 10 dB gains. Therefore, the proposed CIM-GSDM system provides more enhancement than the conventional CIM-SDM system in terms of security of transmission.

**Figure 5:**
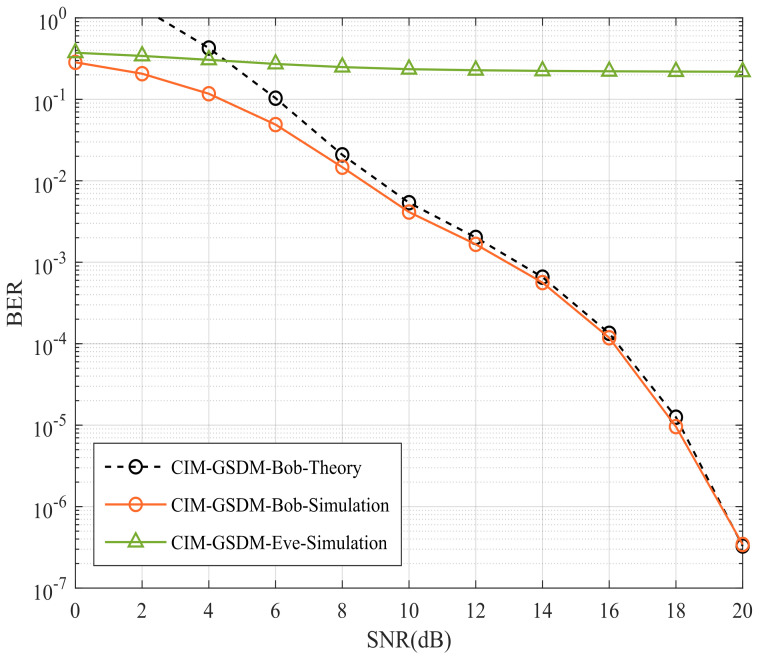
Bob and Eve’s BER performances of proposed CIM-GSDM scheme employing $N_{t}=8$, $N_{r}=8$, $N_{e}=8$ and QPSK.

Figure 5 shows Bob and Eve’s BER performances of proposed CIM-GSDM scheme employing $N_{t}=8$, $N_{r}=8$,$N_{e}=8$ and QPSK. The interference matrix is $\Lambda =diag\{e^{j\Theta_{\Lambda }}\}$$\left(\Theta_{\Lambda }=\left\{28^{{}^{\circ}},32^{{}^{\circ}},40^{{}^{\circ}},45^{{}^{\circ}},50^{{}^{\circ}},55^{{}^{\circ}},60^{{}^{\circ}},65^{{}^{\circ}}\right\}\right)$. From the results, it can be observed that the theoretical results have considerable approximation to the simulation results at high SNR, which convinces that the theoretical analysis are correct. Furthermore, the performance of Eve is much worse than Bob at middle and high SNR even if the receivers at Eve are located near to Bob’s, which means the proposed CIM-GSDM system decreases performance of Eve while keeping Bob unaffected. The theoretical and simulation results match better in high SNR region since the interference caused by noise relatively decreases with SNR becoming higher. As a result, the proposed CIM-GSDM system is able to enhance the security of transmission.

**Figure 6:**
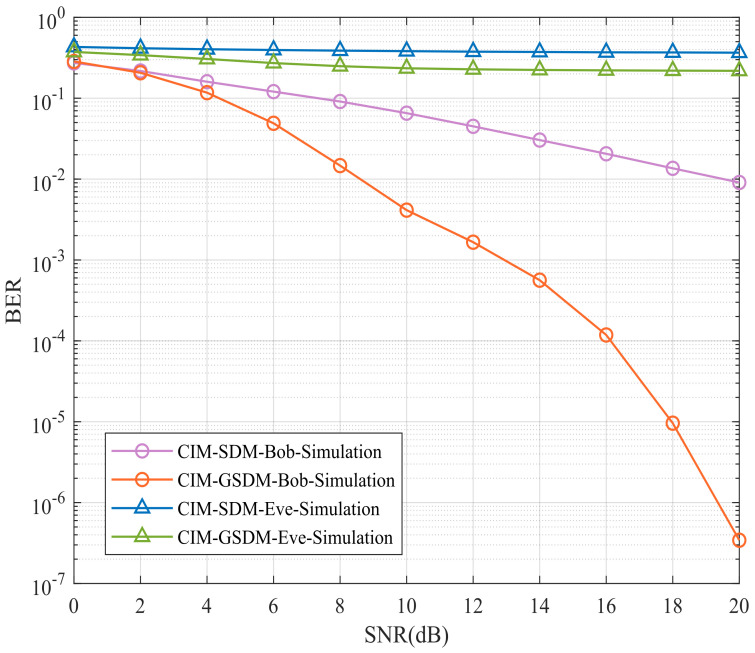
Bob’s and Eve’s BER performances of proposed CIM-GSDM scheme in comparison to the traditional CIM-SDM counterparts employing $N_{t}=8$, $N_{r}=8$, $N_{e}=8$ and QPSK at the same spectral efficiency.

Figure 6 exhibits Bob and Eve’s BER performances of proposed CIM-GSDM scheme in comparison to the traditional CIM-SDM counterparts employing $N_{t}=8$, $N_{r}=8$, $N_{e}=8$ and QPSK at the same spectral efficiency. The results illustrated the proposed CIM-GSDM system enhances the BER performance at both middle and high SNR compared to the original CIM-SDM system at the same spectral efficiency. Analogously, the BER performance of Eve is still worse in both the CIM-GSDM system and the CIM-SDM system even if the receivers at Bob are located near to Bob’s. However, the BER performance of Bob is improved obviously in the CIM-GSDM system, which has almost 12 dB gains. In short, CIM-GSDM system can improve the BER performance strongly at the same spectral efficiency.

## 5. Conclusions

In this paper, a novel CIM-GSDM system is proposed for enhancement of security of transmission in a free space. More specifically, the GSM system is combined with the SDM system for higher transmission rate as a benefit. Furthermore, the design of CIM is taken into consideration as well for the sake of embedding the covert information into the system, which is able to reap their advantages for further improvement of security. Hence, Eve can hardly eavesdrop the transmitted message whether a covert transmission happens. The theoretical analysis of performance of the proposed scheme is also detailed. Our simulation results shows that the BER performance of Bob is much better compared to that of Eve, even if the receivers at Eve are located near to Bob’s. Moreover, the proposed scheme enhances the BER performance compared to original CIM-SDM systems at the same spectral efficiency. In a nutshell, our proposed CIM-GSDM scheme is capable to improve the security of transmission significantly.

## Figures and Tables

**Table 1:** Parameters of simulations.

Figure	Scheme	$N_{t}$	$N_{r}$	$N_{u}$	ΘB	$N_{e}$	ΘE	*M*
Figure 3	CIM-GSDM	8	6	2	$\{15^{{}^{\circ}},60^{{}^{\circ}},120^{{}^{\circ}},$ $160^{{}^{\circ}},215^{{}^{\circ}},285^{{}^{\circ}}\}$	6	$\{30^{{}^{\circ}},75^{{}^{\circ}},135^{{}^{\circ}},$ $175^{{}^{\circ}},230^{{}^{\circ}},300^{{}^{\circ}}\}$	4
Figure 4	CIM-GSDM	8	8	2	$\{15^{{}^{\circ}},55^{{}^{\circ}},90^{{}^{\circ}},130^{{}^{\circ}},$ $170^{{}^{\circ}},210^{{}^{\circ}},250^{{}^{\circ}},290^{{}^{\circ}}\}$	8	$\{30^{{}^{\circ}},70^{{}^{\circ}},105^{{}^{\circ}},145^{{}^{\circ}},$ $185^{{}^{\circ}},235^{{}^{\circ}},265^{{}^{\circ}},305^{{}^{\circ}}\}$	4
Figure 5	CIM-GSDM CIM-SDM	8	6	2	$\{15^{{}^{\circ}},60^{{}^{\circ}},120^{{}^{\circ}},$ $160^{{}^{\circ}},215^{{}^{\circ}},285^{{}^{\circ}}\}$	6	$\{30^{{}^{\circ}},75^{{}^{\circ}},135^{{}^{\circ}},$ $175^{{}^{\circ}},230^{{}^{\circ}},300^{{}^{\circ}}\}$	4
Figure 6	CIM-GSDM CIM-SDM	8	8	2	$\{15^{{}^{\circ}},55^{{}^{\circ}},90^{{}^{\circ}},130^{{}^{\circ}},$ $170^{{}^{\circ}},210^{{}^{\circ}},250^{{}^{\circ}},290^{{}^{\circ}}\}$	8	$\{30^{{}^{\circ}},70^{{}^{\circ}},105^{{}^{\circ}},145^{{}^{\circ}},$ $185^{{}^{\circ}},235^{{}^{\circ}},265^{{}^{\circ}},305^{{}^{\circ}}\}$	4

## Data Availability

Not applicable.
